# Red-Fleshed Apple Flavonoids Extract Alleviates Male Reproductive Injury Caused by Busulfan in Mice

**DOI:** 10.3390/nu15153288

**Published:** 2023-07-25

**Authors:** Bin Wang, Yanbo Wang, Yizhou Chen, Xiaohong Sun, Jihua Xu, Jun Zhu, Yugang Zhang

**Affiliations:** 1 College of Horticulture, Qingdao Agricultural University, Qingdao 266109, China; wb765198@163.com (B.W.); 15820054339@163.com (Y.W.); chenyizhou999@hotmail.com (Y.C.); junzhu@qau.edu.cn (J.Z.); 2 China Engineering Laboratory of Genetic Improvement of Horticultural Crops of Shandong Province, Qingdao Agricultural University, Qingdao 266109, China; 3 College of Life Sciences, Qingdao Agricultural University, Qingdao 266109, China; mingsun9887@163.com (X.S.); xujihua@qau.edu.cn (J.X.); 4 Academy of Dongying Efficient Agricultural Technology and Industry on Saline and Alkaline Land in Collaboration with Qingdao Agricultural University, Dongying 257300, China

**Keywords:** red-fleshed apple, flavonoids, antioxidant, busulfan, reproductive damage

## Abstract

In this research, we analyzed the protective effects of red-fleshed apple flavonoid extracts (RAFEs) on male reproductive injury induced by busulfan, using both in vitro and in vivo models. In the cell-based experiments, RAFEs significantly improved cell viability and proliferation rates compared to control groups. Similarly, in vivo testing with male mice showed that RAFEs and whole apple flavonoid extracts (WAFEs) enhanced various biochemical and liver function-related indicators in the testes; however, RAFEs demonstrated superior efficacy in mitigating testicular damage. Through immunohistochemistry, qRT-PCR, and Western blotting, we found that RAFEs notably enhanced the expression of spermatogenesis-related genes. Moreover, RAFEs increased the expression of oxidative stress- and apoptosis-related genes, thereby effectively reducing oxidative damage in the testes. These findings highlight the potential of RAFEs as natural agents for the prevention and treatment of male reproductive injury, paving the way for future research and potential therapeutic applications.

## 1. Introduction

Infertility is a significant health problem, with 15% of couples facing this issue and more than half of cases attributed to male factors [1]. One of the primary causes of male infertility is sperm disorders such as oligozoospermia [2]. Busulfan is widely used in treating chronic myeloid leukemia and as a pretreatment for hematopoietic stem cell transplantation [3]. However, its side effects have become increasingly evident with its widespread use. Numerous studies have shown that it is highly carcinogenic and teratogenic and can severely affect the functioning of body organs such as the gonads and the central nervous system [4]. In clinical practice, busulfan is often used in combination with cyclophospha mide or fludarabine [5]. Its prolonged use can cause sterility in animals and destroy spermatogonial stem cells in rodents [6]. Histomorphological observations have revealed that the testes of busulfan-injected mice exhibit a significant reduction in size, sperm concentration, and sperm motility; however, in vitro drug intervention within a specific concentration range can recover the damage to the reproductive system [7].

When used in the treatment of young leukemia patients, busulfan is thought to be associated with testicular disorders and atrophy, leading to infertility in many cases. Busulfan-induced germ cell damage in vivo is manifested by upregulating the expression of TNF-α and MCP-1 in mouse support cells and promoting macrophage infiltration into the testes. Studies have shown that damaged sperm plasma induces inflammation of the testicles by activating the expression of TLR2 and TLR4 and inducing the overexpression of inflammatory factors in testis support cells, triggering a non-infectious inflammatory response in the testes [8]. Additionally, the busulfan-treated group showed down-regulated expression of vimentin and ZO-1 in the epididymis at the mRNA and protein levels. Furthermore, total androgen receptor (AR) levels in the testis were increased, and estrogen receptor (ER-α) levels were decreased after busulfan treatment, possibly due to testicular damage [5].

Adipokines affect the body’s energy metabolism and also participate in the regulation of reproductive system function [9]. Both leptin and adiponectin receptors are expressed in testicular tissue, and these two adipokines function in testicular tissue by binding to corresponding receptors [10,11]. Adiponectin is an adipocyte-specific protein that promotes insulin sensitivity and regulates metabolic functions, such as glucose and fatty acid catabolism, while chemokines are considered inhibitors of insulin signaling and glucose catabolism [12]. Leptin, the expression product of the obesity gene code, acts on neurons of the hypothalamic arcuate nucleus, regulates energy metabolism, and keeps body fat relatively stable. Leptin receptors, a product of the diabetes gene code, are located mainly in the hypothalamic arcuate nucleus and the intermediate pathway, which is the center that controls appetite and reproduction. Leptin binds to the receptor and acts on the arcuate nucleus, thus affecting the body’s energy metabolism and reproduction [13].

Reactive oxygen species (ROS) can severely affect oxidative stress development, as evidenced by busulfan’s damage to cells in various organs [14]. Elevated levels of CK-18, a surface marker in Sertoli cells, can also cause impaired spermatogenesis, leading to infertility [14]. Therefore, pharmacological measures are necessary to minimize the adverse effects of busulfan. Many studies have reported reducing busulfan’s harm, such as concurrent use of L-carnitine [15], genistein, dynein lignin [16], and algal oligosaccharides [17]. In recent years, many natural products, such as oligosaccharides, tea polyphenols, and melatonin, have attracted great attention due to their high-quality antioxidant capacity [17]. However, except for red ginseng and olive leaf extracts, plant extracts with antioxidant activity to attenuate busulfan-induced male reproductive system damage in mice or rats have been less studied [18].

Red-fleshed apples originated from Kazakhstan in Central Asia and Ili in Xinjiang, China. This variety’s flowers, fruits, leaves, and branches are red or dark red and rich in anthocyanins [19]. In recent years, red-fleshed apples have been widely used in preparing natural active products due to their richness in various active substances, such as plant polysaccharides, polyphenols, and flavonoids [20]. Flavonoids have a wide range of biological functions, and their biological activities have been extensively studied by biologists. Numerous studies have demonstrated that flavonoid products can significantly mitigate elevated intracellular free radicals and are effective in maintaining cellular vital signs [21]. For instance, Icariin I, a novel anticancer drug isolated from Epimedium, can significantly inhibit the growth of B16F10 melanoma in vivo by modulating intestinal flora and host immunity [22]. Green tea flavonoids have been reported to inhibit the growth of Streptococcus suis, reduce pathogenicity, and positively affect pyrimidine metabolism and protein digestion and absorption [23]. Flavonoids in green tea significantly alleviate kidney stone injury in rats and modulate oxidative stress pathways [24]. Researchers conducted chemical studies on the above-ground parts of Andrographis paniculata and isolated nine compounds, verifying that they have pro-apoptotic effects on cancer cells such as LNCaP and HepG2 and could significantly inhibit the overproduction of nitric oxide in macrophages [25]. In red-fleshed apples, the primary anthocyanin species is cornflower, which can reduce the area of atherosclerotic blockage and the vascular-dependent diastolic response induced by a high-fat, high-cholesterol diet in mice [26]. Thus, flavonoids are essential as natural antioxidants in preventing cancer, boosting immunity, and balancing blood glucose levels [27].

Red-fleshed apples are rich in flavonoids and other natural active ingredients and have excellent potential for food and health applications. However, there is limited research on red-fleshed apples’ health functions and related mechanisms in alleviating reproductive damage. In this study, we analyzed the function and mechanism of red-fleshed apple flavonoids in alleviating leucovorin-induced spermatogonia damage in mice and male reproductive damage in mice, using red-fleshed apple resources created and collected by the research team as test materials. This study provides a theoretical reference for the in-depth exploration and evaluation of the health functions of red-fleshed apples, the creation and utilization of new apple germplasm, and the breeding of more superior functional apple varieties.

## 2. Materials and Methods

### 2.1. Experimental Design of the Study

Previous studies have shown that flavonoids present in red-fleshed apples can repair damage to spermatogenesis caused by busulfan. Here, we investigated the correlation between red-fleshed apple flavonoids and spermatogenesis through immunohistochemical assays and other biological means, using both in vitro spermatogenic cell models and in vivo organ damage models.

### 2.2. Cell Experimental Protocol

We cultured type A spermatogonia (GC-1 cells) in Dulbecco’s modified Eagle medium/nutrient blend F12 (DMEM/F12, Gibco, Grand Island, NY, USA). The medium was supplemented with 2 mM L-glutamine (Invitrogen, Carlsbad, CA, USA), 10% fetal bovine serum (FBS, Gibco), and 100 units/mL penicillin and streptomycin (Invitrogen). The cells were cultured in a humidified incubator at 37 °C with 5% CO_2_. Subculturing was performed every three days, and cell dissociation was treated with 0.25% trypsin and EDTA (Invitrogen) before reseeding into six-well cell culture plates for subsequent experiments. The medium was changed every two days. RAFEs were dissolved to 10^−7^ M with 1% ethanol saline, and busulfan with DMs was varied from 0 to 10^−4^ μM. Cells were treated with busulfan (Busulfan), busulfan and RAFEs (BF2, BF5, BF10) for 24 h before the experiments.

### 2.3. Breeding Environment of Mice

Male ICR mice were obtained from Beijing Vital River Laboratory Animal Technology Co., Ltd. (Beijing, China), and housed in a room with a constant temperature of 22–23 °C and a 12-h light-dark cycle. The mice had free access to food and water. The study was approved by the Animal Care and Ethics Committee of Qingdao Agricultural University and was conducted in accordance with the National Institutes of Health (NIH) guidelines for the care and use of laboratory animals (NIH Publications no. 8023).

### 2.4. Preparation and Extraction of Red-Fleshed Apple Flavonoids Extract (RAFEs)

Thin slices of red-fleshed apples were frozen quickly and ground into a powder using liquid nitrogen. The powder was then extracted using 0.1% phosphoric acid/ethanol in a 1:30 ratio. The extract was sonicated for 30 min and left to stand in the dark for 12 h. The filter residue was separated from the extract, which was then subjected to a second extraction using the same procedure as before. After the second extraction, the two extracts were combined, sonicated, and left to stand for 12 h. The resulting extract was concentrated using a nitrogen-blowing meter until it became viscous and hung from the device. The concentrated extract was then fixed to a specific volume using deionized water. The red-fleshed apple flavonoid extract was subsequently extracted three times with equal amounts of petroleum ether and ethyl acetate. The resulting flavonoid extract was freeze-dried for 48 h to remove the residual aqueous phase. After the initial extraction, we selected NKA-9 macroporous adsorption resin for purification and preparation. Before filling the column, the resin was pickled and alkali washed, and then washed with distilled water to a pH of 7. The flavonoid extract was 20 mL, and the loading flow rate was controlled at 1 mL/min. After the resin had absorbed and balanced the flavonoids, the impurities were washed away by deionized water and then eluted at the same flow rate with different concentration gradients of ethanol eluents. All eluents were collected and then concentrated and dried to a certain volume with a nitrogen blower and a vacuum freeze dryer. Then high-performance liquid chromatography was performed.

### 2.5. Treatment of Mouse

Many recent studies of building sterile animal models have utilized busulfan as a treatment [20]. The preparation of RAFEs has been previously described in our publication [28]. In this study, we investigated the mitigating effect of RAFE on busulfan-induced mouse spermatozoa and its underlying mechanism. The treatment group comprised mice that received 40 mg/kg busulfan and 1–10 mg/kg RAFEs via oral gavage.

Fresh solutions of RAFEs were prepared using ultrapure water. Ten different treatment groups with ten mice (three weeks of age) were randomly assigned to each group, including: (1) control group (ultrapure water gavage), (2) 1 mg/kg RAFEs control group (CF1), (3) 1 mg/kg WAFEs control group (CWF1), (4) busulfan pathology group (Busulfan), (5) busulfan + low-dose RAFEs (2 mg/kg) treatment group (BF2), (6) busulfan + medium-dose RAFEs (5 mg/kg) treatment group (BF5), (7) busulfan + high-dose RAFEs (10 mg/kg) treatment group (BF10), (8) busulfan + low-dose WAFEs (2 mg/kg) treatment group (BWF2), (9) busulfan + medium-dose WAFEs (5 mg/kg) treatment group (BWF5), and (10) busulfan + high-dose WAFEs (10 mg/kg) treatment group (BWF10). The liquid volume for daily gavage was 0.10 mL per mouse, and the experiment was conducted for five weeks with daily tube feeding. After five weeks, the testes, blood, and sperm were collected from the sacrificed mice for subsequent analysis.

### 2.6. Flow Cytometry Assay

Cell cycle analysis was performed using propidium iodide (PI) staining. Adherent cells were digested with 0.25% trypsin and washed with phosphate-buffered saline (PBS) prior to flow cytometry analysis. The samples were stained with 50 µg/mL PI and 50 µg/mL RNase A for 20 min while maintaining a temperature of 37 °C during the staining process (Altra; Beckman Co., Brea, CA, USA).

Apoptosis was evaluated using annexin V-FITC and PI staining. The cells were gently resuspended in 500 µL of PBS, and 5 µL of annexin V-FITC and 5 µL of PI were added to the medium and mixed well. The cells were then incubated at room temperature in the dark for 10 min before FACS analysis. Intracellular Ca^2+^ levels were measured using Fluo-3 AM (Beyotime, China). The cells were pretreated with busulfan or 4pBA for 24 h, loaded with Fluo-3 AM for 1 h, and analyzed by flow cytometry after treatment.

### 2.7. Evaluation of Sperm Motility and Concentration

The analysis of sperm quality starts at the same time as the sample collection. Epididymal sperm were cultured in DMEM/F12 medium (Gibco, 8119172, USA) supplemented with 10% FBS (Gibco, 10099-141, USA). The medium was incubated on a heated stage at 37.5 °C for 5 min after preparation. The sample was placed in the counting chamber, and sperm motility and concentration were determined using an SCA sperm analyzer [29]. Sperm motility was evaluated using WHO criteria: grade A for sperm with a curvilinear velocity above 22 µm/s; grade B for sperm with a curvilinear velocity between 5 µm/s and 22 µm/s; grade C for sperm with a curvilinear velocity below 5 µm/s; and grade D for immotile spermatozoa [30]. Sperm concentration was assessed by diluting sperm with a medium during motility analysis [30].

### 2.8. H&E and Immunofluorescence

After sacrificing the mice, the testicles were fixed with 4% paraformaldehyde and dehydrated with different concentrations of organic solutions. The samples were then embedded in paraffin blocks and cut into 5 µm sections using standard histological observation procedures. The prepared paraffin sections were stained with H&E following previous procedures [20]. The testicular paraffin sections were also used for immunohistochemistry, but antigen retrieval was required after removing the organic solution. The sections were blocked with a blocking buffer for 30 min, followed by incubation with primary (Supplementary Table S1) and secondary antibodies (Beyotime, A0516, Nantong, China). The sections were then observed and imaged under a fluorescence microscope (Olympus, BX51, Tokyo, Japan).

### 2.9. TUNEL Assay of Apoptosis in Mouse

The collected testicular tissue was fixed in 4% paraformaldehyde and washed twice with PBS. The treated tissue was sliced and incubated with a terminal deoxynucleotidyl transferase dUTP nick end labeling (TUNEL) working solution under dark conditions. After stopping the reaction, 4′,6-diamidino-2-phenylindole dihydrochloride (DAPI) solution was added and incubated at room temperature in the dark. The slides were covered, and three random fields of view were observed under a fluorescence microscope.

### 2.10. qRT-PCR

After extracting the total RNA, reverse transcription was performed according to the kit instructions [TransScript One-Step gDNA Removal Kit and cDNA Synthesis Kit (TransGen, AT311-03, Beijing, China)]. To determine changes in expression levels of different mRNAs in the testes, we performed quantitative real-time PCR (qRT-PCR) using the transcribed cDNA. The qRT-PCR instrument used was a LightCycler 480 real-time PCR instrument (Roche, Basel, Switzerland), and the samples on board were prepared using the LightCycler SYBR Green I Master Mix according to the manufacturer’s instructions. The reaction system in this study consisted of 24 μL, containing 2 μL of cDNA, 12 μL of SYBR green master mix, 1.2 μL of the primer mixture (containing forward and reverse primers), and 8.8 μL of RNase-free water. The gene primer sequences are listed in Supplementary Table S2.

### 2.11. Western Blot Analysis

The steps for extracting testicular protein were as follows: samples were treated with pre-chilled radioimmunoprecipitation assay (RIPA) buffer and subjected to cryogenic disruption, followed by centrifugation at 10,000× *g* for 10 min to collect the supernatant. Sodium dodecyl sulfate (SDS) loading buffer was added to the supernatant, and the mixture was boiled in water for 5 min. The denatured proteins were then loaded onto an electrophoretic gel and processed at 100 V for 1.5 h. The proteins on the gel were transferred to a polyvinylidene fluoride membrane. The membrane was then blocked with bovine serum protein at low temperature for more than 1 h, followed by washing three times with Tris buffer and 0.1% Tween 20 (TBST). The membrane was then incubated overnight at 4 °C with a diluted primary antibody. The next day, the membrane was washed three times with TBST and incubated with HRP-labeled secondary antibodies (Beyotime, A0208, Nantong, China) at room temperature for 1 h. The membrane was then washed three times with TBST, imaged with developer solution, and observed.

### 2.12. Statistical Analysis

All experiments in this study had more than three replicates, and the results were expressed as the mean ± standard deviation (SD). The software used for data analysis was Prism. The analysis methods used were one-way analysis of variance (ANOVA) and multiple comparisons of LSD. A *p*-value of less than 0.05 was considered statistically significant, while a *p*-value of less than 0.01 was considered highly significant.

## 3. Results

### 3.1. RAFEs Restore Spermatogonia Viability in Mouse

**Figure 1:**
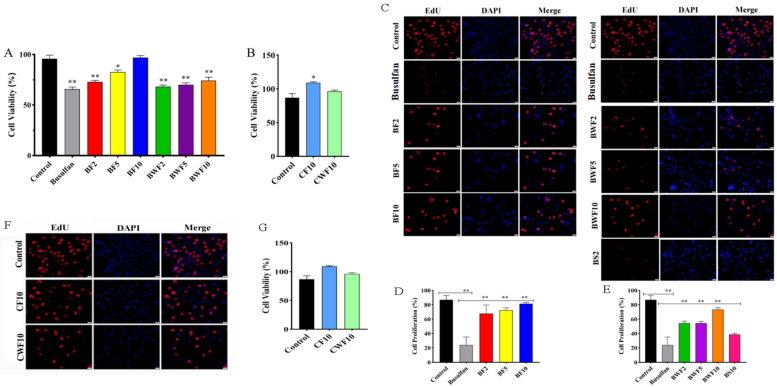
Effects of red-fleshed apple flavonoids extract (RAFEs) on mouse spermatogonial cells: (**A**) the restorative effect of RAFEs and WAFEs on injured mouse spermatogonial cells; (**B**) the health effects of RAFEs and WAFEs on normal mouse spermatogonial cells; (**C**,**D**) RAFEs improves the proliferation of injured spermatogonial cells in mice; (**C**,**E**) effect of WAFEs on the proliferation of injured spermatogonial cells in mice; (**F**) EdU staining showed cell proliferation effect; (**G**) positive cell rate statistics. The results are presented as mean ± SD. * means 0.01 < *p* < 0.05 and ** means *p* < 0.01.

The effect of different concentrations of RAFEs and WAFEs on spermatogonia cell viability after busulfan-induced damage in mice was measured using the CCK8 kit. The results showed that both RAFEs and WAFEs were effective at restoring spermatogenic cell injury in mice. Overall, the recovery effect of RAFEs was greater than that of WAFEs, with the BF10 group showing the most significant recovery effect, which was almost indistinguishable from the control group (Figure 1A). The above results tentatively prove that RAFEs have a significant recovery effect on spermatogonia damage caused by BF10 in mice.

Next, normal mouse spermatogonia were treated with RAFEs and WAFEs. The results demonstrated that the performance of each treatment group was superior to that of the control group, indicating that the effect of RAFEs was significant, with cell viability reaching 131.77% (Figure 1B).

The EdU staining results showed that the EdU positive rate of cells treated with RAFEs (BF2, BF5, BF10) and WAFEs (BWF2, BWF5, BWF10) was significantly higher than that of the busulfan group (Figure 1C), and the recovery effect was highly significant in the BF10 group, with a positive cell rate of 91.34%. Furthermore, the viability of mouse spermatogonia treated with the same concentration of the centaureidin-3-O-galactoside standard (BS10) was significantly restored to 54.32%. However, its restoration effect was less effective than that of the BF10 group (Figure 1D,E).

Moreover, healthy and cell-viable mouse spermatogonia were treated with the same concentration of RAFEs and WAFEs, respectively. The EdU results demonstrated that RAFEs had a significant proliferation-promoting effect on spermatogonia (Figure 1F,G), while WAFEs did not have a significant proliferation effect on mouse spermatogonia.

**Figure 2:**
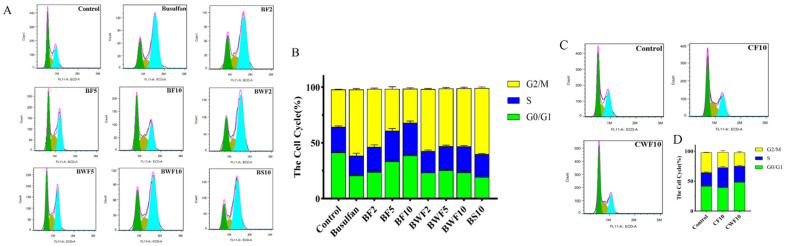
Cell cycle detection: (**A**) effect of RAFEs and WAFEs on cell cycle of injured mouse spermatogonial cells; (**B**) statistical analysis of each cycle of spermatogonial injury; (**C**) effects of RAFEs and WAFEs on cytologic changes of normal mouse spermatogonial cells; (**D**) statistical analysis of each cycle of spermatogonial cells.

The results of cell cycle detection by flow cytometry showed that the number of spermatogonia in the S-phase was 22.41%, 27.48%, and 27.94% in the BF2, BF5, and BF10 groups, respectively, which was higher than the 17.52% in the busulfan group (Figure 2A,B). All the above results indicated that RAFEs could promote the proliferation of spermatogonia in mice. Consequently, flavonoid intervention could effectively alleviate the cell cycle imbalance caused by busulfan.

During the cell cycle monitoring of the health treatment group, it was discovered that both WAFEs and RAFEs positively affected normal mouse spermatogonia. The number of cells in the S-phase was 26.66% and 33.11% for WAFEs and RAFEs, respectively, which was significantly greater than the control group’s value of 22.66% (Figure 2C,D).

### 3.2. Effects of RAFEs on Weight of Mice Injected with Busulfan

**Figure 3:**
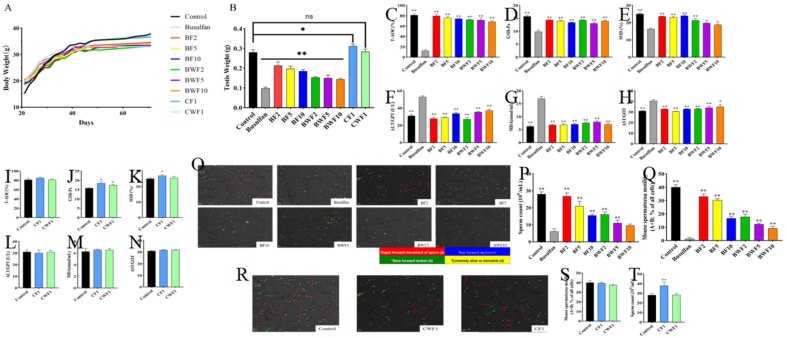
RAFEs ameliorated reproductive organ injury induced by busulfan in mice. (**A**) Effects of RAFEs on body weight (**B**) and testicular weight (**C**) in mice, total antioxidant capacity (T-AOC), (**D**) glutathione peroxidase (GSH-PX), (**E**) SOD, (**F**) alanine aminotransferase (ALT), (**G**) MDA, and (**H**) aspartate aminotransferase (AST) in mice with busulfan-induced reproductive injury. (**I**) Effects of RAFEs and WAFEs on plasma total antioxidant capacity (T-AOC), (**J**) glutathione peroxidase (GSH-PX), (**K**) SOD, (**L**) alanine aminotransferase (ALT), (**M**) MDA, and (**N**) aspartate aminotransferase (AST) in mice. (**O**–**Q**) Effects of RAFEs and WAFEs on sperm in mice with reproductive injuries. (**R**–**T**) Effects of RAFEs and WAFEs on normal mouse sperm. The results are presented as mean ± SD. * indicates 0.01 < *p* < 0.05 and ** indicates *p* < 0.01.

Before treatment, the average weight of the mice in each group was approximately 19 g, and there were no health issues. The mice were weighed every two days, and the weight of each group increased steadily until the mice were five weeks old. After this time, the weight of the mice plateaued, and there was no significant weight change (Figure 3A). After five weeks of gavage treatment, the mouse testes were dissected and weighed. It was discovered that the testes’ weights were significantly lower in the BWF1, BF3, and BF5 groups compared to the control group (0.278 g). However, the BWF1 group exhibited a recovery effect (Figure 3B).

### 3.3. RAFEs Modifies Biochemical Parameters in Mice

The plasma T-AOC of mice in the control group was 80.4%, and the antioxidant capacity of the busulfan group was significantly lower than that of the control group. After intervention with RAFEs, the treatment groups at different concentrations showed significant relief, with the BF2 group (2 mg/kg/day) restoring the total antioxidant capacity to 80%, which was almost the same as the control group (Figure 3C).

Antioxidant enzymes [(Superoxide Dismutase (SOD) and Glutathione Peroxidase (GPx)] protect against oxidative stress in vivo. The depletion of antioxidant substances such as SOD, GPx in plasma, infiltration of inflammatory cells into organ cells, and activation of leukocytes to produce large amounts of free radicals when viruses attack internal organs usually cause damage to peroxides in the body. Peroxidases and non-enzymatic free radical scavengers are responsible for scavenging free radicals in the body. A decrease in the activity and levels of peroxidase scavengers leads to a decrease in the body’s antioxidant capacity, resulting in the accumulation of free radicals in the body.

Compared to the busulfan group, there were significant improvements in all other treatment groups, with BF1 exhibiting the best result (Figure 3D,E). The levels of SOD and GSH-Px were restored in all treatment groups, indicating that flavonoids can improve busulfan-induced testicular oxidative stress and alleviate oxidative damage in vivo. RAFEs were found to be effective in alleviating liver damage in mice by testing liver damage indicators (ALT, AST). The process of lipid peroxidation is determined by malondialdehyde (MDA). The experimental results showed that there was serious damage to the biofilm after busulfan intervention, which resulted in a significantly higher content of MDA than that of the control group. However, the content of each treatment group was significantly reduced, indicating that flavonoids could effectively reduce biofilm damage (Figure 3F–H). The results of plasma biochemical indexes in the healthcare group showed that both RAFEs and WAFEs had certain healthcare effects, and the effect of RAFEs was better than that of WAFEs (Figure 3I–N). The biochemical indexes of plasma showed that RAFEs significantly improved busulfan-induced oxidative stress in mice and helped to reduce oxidative damage in mice.

### 3.4. RAFEs Increases Sperm Motility and Sperm Concentration

Sperm concentration is an effective indicator for the development of the epididymis and the health of sperm in mice. The sperm distribution in the control group was tightly distributed, and the highest sperm concentration was 37.2 million/mL. In contrast, the epididymis of the busulfan group was almost devoid of sperm. Compared to the busulfan group, RAFEs and WAFEs had a significant recovery effect on mouse sperm motility (sum of the percentages of class a + class b) after treatment, with the BF2 and BF5 groups exhibiting the most significant recovery effect (Figure 3O,P) (*p* < 0.01). Figure 3Q also displayed a significant recovery in mouse sperm concentration after RAFEs treatment (*p* < 0.01).

Gavage treatment of normal mice with flavonoids did not result in significant changes in sperm viability. However, the sperm concentration in CF1 was significantly higher than in normal mice (Figure 3R–T).

### 3.5. Effects of RAFEs on Busulfan-Induced Damage to Different Tissues

**Figure 4:**
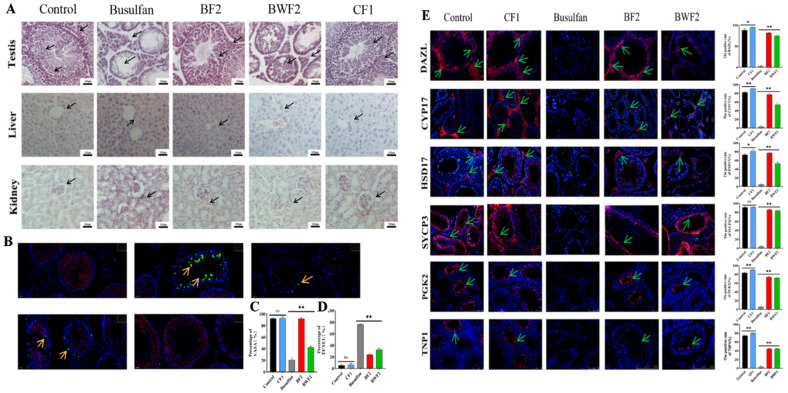
RAFEs increased the germ cell quantity. (**A**) Effects of RAFEs and WAFEs on testis, liver, and kidney in mice: (**B**) VASA co-infection with TUNEL; (**C**) VASA positive cell rate statistics; and (**D**) TUNEL positive cell rate statistics. Histopathology photos of HE staining and MVH and DAZL staining of mouse testes. Immunohistochemistry for some of the marker genes. The results are presented as mean ± SD. “ns” stands for no difference, * indicates 0.01 < *p* < 0.05, and ** indicates *p* < 0.01. Note: (**A**): In the testicular section, the black arrow indicates primary/secondary spermatocytes. In the liver section, the black arrow indicates cell necrosis. In kidney slides, black arrows indicate damage to glomerulus. (**B**) Yellow arrows show apoptotic cells. (**E**) Green arrow indicates primary/secondary spermatocytes, spermatids, and spermatozoa.

The HE staining of mouse livers (Figure 4A) revealed that spermatogenic tubules in the control group grew normally, with full and tightly arranged cells. However, in the busulfan group, the cells inside the spermatogenic tubules were seriously damaged, showing cavitation, flocculation and other symptoms of damage. These results indicate that busulfan treatment caused serious damage to mouse testes. In addition, compared with the control group, the diameter of the seminiferous tubule was significantly reduced after treatment. In the BF2 group, the cross section diameter of the tubule of the mouse testes had an obvious recovery effect, and the cells in different developmental stages of the tubule were closely arranged, basically no different from the control group. HE staining results of BWF2 group showed that white meat apple flavonoid extract also had a significant recovery effect on mouse testicular tissue and could significantly alleviate the damage of the seminiferous tubules, but the cells in the seminiferous tubules were sparsely arranged. The diameter of the fine tubule in CF1 group was no different from that in the control group, and the internal cells were full, which was a landmark sample of healthy testes. In RAFEs-treated mice, liver cells were tightly arranged and well-developed in the central vein, suggesting that flavonoids alleviated liver damage caused by busulfan. In mouse renal pathological observation, it was discovered that the glomerular capillary bulbs in the busulfan group were loosely lobed, and the cells were sparsely arranged and disorderly. Interestingly, after flavonoid intervention, the kidney cells were neatly arranged. We further quantitated the related parameters in seminiferous tubules in the control and treatment groups (Table 1). After the busulfan treatment, the number of spermatogonia, spermatocytes, and Leydig cells, and the diameter of seminiferous tubules were significantly decreased (Busulfan, *p* < 0.01); while in CF1 and BF2, the value of all these four parameters was significantly increased when compared with busulfan (*p* < 0.01). However, in BWF2, changes in the number of spermatogonia and Leydig cells were not significant when compared with BA0 (Table 1).

### 3.6. RAFEs Improves the Expression of Important Genes Involved in Spermatogenesis

The process of spermatogenesis is complex and regulated by numerous genes, such as DDX4 (VASA), CREM, SYCP3, DAZL, GDNF, PGK2, KIT, KITLG, MSH4, MSH5, SNAI3, and ZFP37. To further validate the efficacy of RAFEs against male reproductive damage induced by busulfan, we utilized immunohistochemistry to detect the spatial expression of several gene products in mouse testes (Figure 4B). The results revealed that VASA expression was significantly decreased in the busulfan group (*p* < 0.01), while a significant alleviating effect was observed in all treatment groups (Figure 4C) (*p* < 0.01). The TUNEL analysis results displayed that almost no apoptotic cells were observed in the control group, but the apoptosis rate increased significantly after busulfan intervention. Interestingly, after the intervention of RAFEs, the degree of cell apoptosis decreased (Figure 4B,D) (*p* < 0.01).

A decrease in the number of protein-positive cells may indicate disruption of the spermatogenesis process. The immunohistochemistry results for DAZL, CYP17, and HSD17 showed a significant reduction in the percentage of positive cells after busulfan intervention compared to the control group, while the percentage of positive cells for BF2 and BWF2 displayed significant recovery (*p* < 0.01) (Figure 4E). In mice, PGK2 was highly expressed at the primary spermatocyte stage, and the trend of positive cell recovery in its treatment group was essentially the same as that of TNP1.

SYCP3 was primarily expressed in primary spermatocytes, and its role in meiosis was highly significant in the BF2 group (*p* < 0.01) and almost indistinguishable from that of the control group. SYCP3 was also highly expressed in mesenchymal cells (Figure 4E). Compared to the control group, protein expression in the CF1 group was significantly increased, except for SYCP3 (Figure 4E).

**Figure 5:**
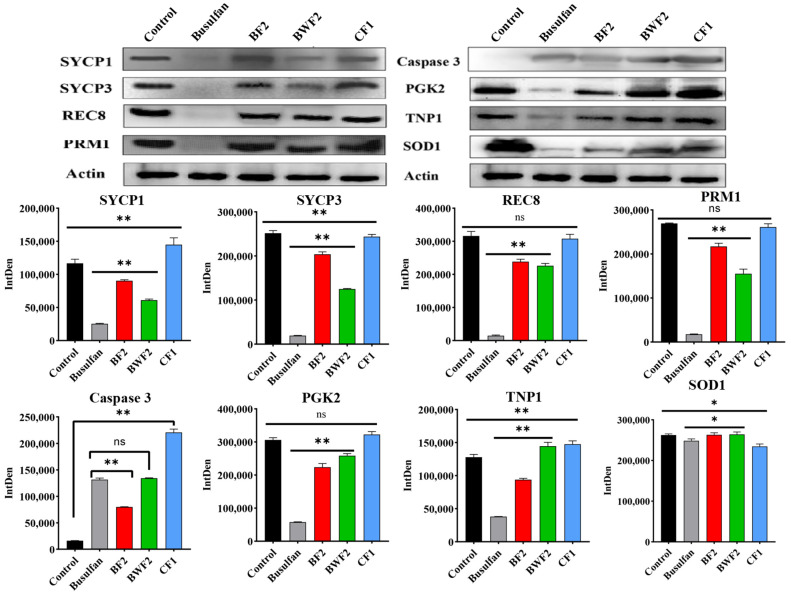
The expression of important proteins in spermatogenesis. The results are presented as mean ± SD. “ns” indicates no difference, * indicates 0.01 < *p* < 0.05, and ** indicates *p* < 0.01.

**Figure 6:**
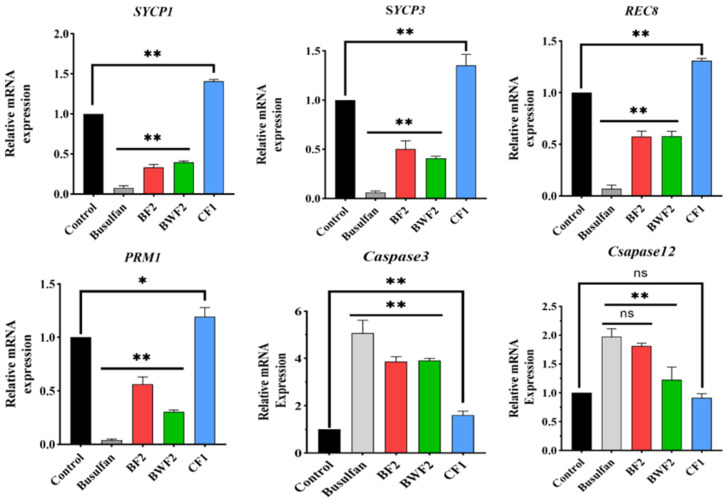
The expression of important genes in spermatogenesis by q-RT-PCR. The results are presented as mean ± SD. “ns” indicates no difference, * indicates 0.01 < *p* < 0.05, and ** indicates *p* < 0.01.

To investigate the protective mechanism of RAFEs against the toxic effects of busulfan, Western Blot (Figure 5) and qRT-PCR (Figure 6) studies were conducted on testicular tissues from different treatment groups of mice. Hormones such as testosterone and estrogen play a crucial role in spermatogenesis, while levels of the proteins PRM1 and REC8, which are essential for synthesizing these hormones, increased after the RAFEs intervention. Compared to the control group, the expression of the PRM1, REC8, SYCP1, and SYCP3 genes in the busulfan group significantly decreased (*p* < 0.01), which was consistent with the qRT-PCR results (Figure 6).

SOD1 expression was significantly reduced in the white elimination group, and PGK2 and TNP1 showed a similar expression trend. There was some recovery in both RAFEs and WAFEs (Figure 5). qRT-PCR results also showed a similar trend (Figure 6). The data suggest that RAFEs facilitate the creation of a testicular microenvironment, thereby improving spermatogenesis. The Western Blot and qRT-PCR assays revealed that the treatment of the health care group (CF1) significantly promoted the health of the testicular microenvironment in mice.

## 4. Discussion

Natural active substances are gaining wider acceptance due to the increased interest in food safety in recent years [31]. Previous studies using pomegranate, purple cauliflower, and purple cabbage have shown that flavonoids have a strong ability to scavenge free radicals [30]. In recent years, functional foods rich in flavonoids have received much attention for their beneficial effects [32]. One study found that phenolic compounds extracted from blackberries are growth-inhibiting compounds with anticancer activity [33]. Another study indicated that the intake of anthocyanins could effectively prevent or improve visual fatigue that occurs after work [34]. The same paper states that anthocyanins alleviate or prevent severe myopia and other eye diseases by regulating key genes associated with the normal development of retinal cells. Additionally, anthocyanins can effectively alleviate alcoholic liver injury because they significantly reduce the release of alanine aminotransferase in hepatocytes and restore redox balance [35].

Studies using anthocyanins extracted from red-fleshed apples to treat porcine ovarian granulosa cells have shown that anthocyanins extracted from the peel in red-fleshed apples have a mitigating effect on oxidative damage in porcine granulosa cells [36]. Additionally, red-fleshed apples may inhibit the proliferation of human breast cancer, possibly due to their antioxidant and antiproliferative properties [37]. Although there have been several studies on the functional analysis of active substances in apples, there have been no reports on restoring damage to mouse spermatogonia by red-fleshed apple flavonoids. In this study, mouse spermatogonia were selected as a model for in vitro testing, and it was found that spermatogonia treated with busulfan showed recoverable functional damage. After treatment with RAFEs, busulfan-induced damage to mouse spermatogonia showed significant recovery, with the BF10 group showing the best recovery effect. We observed the morphology and cell cycle of RAFEs-treated damaged mouse spermatogonia using EdU fluorescent labeling and flow cytometry. We found that spermatogonia showed significant recovery and stable cell cycle recovery, indicating that RAFEs had a significant restorative effect on busulfan-induced damage to mouse spermatogonia.

Several studies have reported various methods to reduce the side effects of busulfan on male reproduction, particularly during spermatogenesis [38]. Molybdenum rescues germ cell development and maintains blood levels of testosterone, such as estradiol and luteinizing hormone, to improve spermatogenesis in mice [39]. Olive leaf extract has been found to ameliorate the negative effects of busulfan on spermatogenesis [18], while Gallus has been found to attenuate busulfan-induced spermatogenesis disorders. In addition, Chi et al. reported that genistein reduced intra-testicular testosterone (ITT) levels and improved spermatogenesis in rats after busulfan intervention [16], suggesting that it could preserve male fertility in cancer patients treated with busulfan. Some studies have found that red-fleshed apples contain a high concentration of flavonoids, which are effective in scavenging various free radicals and have significant antioxidant activity [30]. Levels of ROS in oocytes and spermatogonia are closely associated with apoptosis [40].

To explore the mechanism of action of RAFE in alleviating reproductive injury in mice after busulfan intervention, we verified its effect on the recovery of reproductive damage in male mice by observing histological structures, immunohistochemistry, Western Blot, qRT-PCR, and other physiological and molecular biological assays. We found that RAFEs effectively alleviated testicular cell damage caused by busulfan, eliminated excess intracellular ROS, and inhibited multiple apoptotic signaling pathways. In the histological structure of the testis, we found that after treatment with RAFEs, the cells in the testicular varicose ducts were tightly arranged, and the cell development in all parts of the testes returned to normal. This restorative effect was observed by observing sperm vitality and sperm concentration in mice. Additionally, busulfan has some damaging effects on the liver and kidneys, and RAFEs can also alleviate these effects. The total antioxidant capacity of the flavonoid treatment group was significantly enhanced, with the BF1 group almost returning to the normal group level. Furthermore, after flavonoid intervention, some basic biochemical indicators also returned to normal levels, such as ALT, AST, and T-SOD, which are important indicators to judge the level of active samples of organisms.

Furthermore, the decrease in the number of positive cells may be due to the disruption of the spermatogenesis process induced by busulfan, and changes in the expression of key genes and proteins may be the major reason for RAFE to restore male germ cells. To test this hypothesis, we chose to verify the expression of certain key genes during spermatogenesis, such as DDX4, PGK2, and TNP1, key genes during meiosis, such as PRM1, REC8, and SYCP3, as well as apoptosis and oxidative stress-related genes, such as Caspase3 and SOD1 [41]. Men with hypospermia have very low levels of mRNA and protein expression in VASA, which suggests that VASA expression levels may be related to the onset of male infertility, and this finding can be used as a molecular marker for diagnosing male infertility [42]. In the testicular tissues of mice treated with busulfan, several genes involved in spermatogenesis showed a similar trend of change, indicating that busulfan interferes with the spermatogenesis process by regulating genes.

However, after treatment with flavonoids, the expression of each marker gene returned to levels almost similar to those of the control group. One study suggested that fucoidan oligosaccharides can reduce busulfan-induced reproductive damage by increasing the level of expression of TP1 marker genes [17]. Our results showed that busulfan interfered with the process of spermatogenesis, whereas RAFEs protected spermatogenesis by alleviating intracellular oxidative stress damage and scavenging excess free radicals, thereby restoring the developmental process of spermatogonia. We then analyzed the protein expression of each of these key genes and found that busulfan significantly reduced the protein levels of SYCP1, SYCP3, and PRM1, genes involved in spermatogenesis, while RAFEs increased their protein levels. These genes are involved in the synthesis of hormones that are essential for spermatogenesis, such as testosterone and estrogen. In addition, there was a significant increase in the expression of the Caspase3 gene, an apoptotic gene, and the protein expression level was significantly reduced and normalized after flavonoid treatment. Furthermore, qRT-PCR of some key genes in spermatogenesis revealed that the expression trend of each gene was generally consistent with protein expression.

These data suggest that flavonoids can improve the testicular microenvironment to enhance the spermatogenesis process by promoting the expression levels of genes and proteins associated with spermatogenesis. Thus, this study preliminarily proves that RAFEs can alleviate the reproductive damage induced by busulfan in male mice by regulating the expression of some key genes related to spermatogenesis. This finding provides a theoretical basis and reference value for breeding functional red-fleshed apples.

## Figures and Tables

**Table 1:** Quantification of related parameters in seminiferous tubules.

Group	Control	CF1	Busulfan	BF2	BWF2
Spermatogonia	39.42 ± 3.42 **	42.49 ± 4.32 **	18.38 ± 2.11 **	35.22 ± 2.13 **	25.49 ± 2.40
Spermatocytes number	164.24 ± 9.73 **	173.39 ± 10.48 **	38.58 ± 4.42 **	143.49 ± 8.82 **	69.32 ±4.29 *
Leyding cells number	24.31 ± 2.13 **	28.59 ± 3.98 **	7.24 ± 0.59 **	21.32 ± 1.34 **	10.46 ± 0.83
Seminiferous tubules diameter	231.42 ± 6.42 **	252.38 ± 7.53 **	98.49 ± 2.49 **	194.47 ± 5.42 **	132.42 ± 2.68 **

Within the samples, significant differences * *p* < 0.05 and ** *p* < 0.01, respectively. The data were presented as mean ± SD.

## Data Availability

All data generated or analyzed for this study are included in this published article and its supplementary information files.

## Supplementary Materials

The following supporting information can be downloaded at: https://www.mdpi.com/article/10.3390/nu15153288/s1, Table S1: The primary antibodies information. Table S2: The qRT-PCR primers sequences and the length of the amplified products.
