# Supplementary material for: Red-Fleshed Apple Flavonoids Extract Alleviates Male Reproductive Injury Caused by Busulfan in Mice
Source: Nutrients. 2023 Jul 25;15(15):3288. doi: 10.3390/nu15153288 (PMC10420934; doi:10.3390/nu15153288)
Supplement: Supplementary file 1 [file nutrients-15-03288-s001.zip › nutrients-2458483-supplementary.pdf]

Table S1. The primary antibodies information.

Antibody	Corporate Brand	Cat. #	Immusourcer
VASA	Sangon Biotech (Shanghai) Co., Ltd.	D161611	Rabbit (polyclonal)
PGK2	Abcam	ab186742	Rabbit (polyclonal)
TNP1	Abcam	ab73135	Rabbit (polyclonal)
DAZL	Abcam	ab215718	Rabbit (polyclonal)
CYP17	Beijing Biosynthesis Biotechnology CO.	bs-6695R	Rabbit (polyclonal)
HSD17	Beijing Biosynthesis Biotechnology CO.	bs-23342R	Rabbit (polyclonal)
SYCP1	Beijing Biosynthesis Biotechnology CO.	bs-17297R	Rabbit (polyclonal)
SYCP3	Sangon Biotech (Shanghai) Co., Ltd.	D162171	Rabbit (polyclonal)
REC8	Abcam	ab192241	Rabbit (polyclonal)
PRM1	Abcam	ab66978	Rabbit (polyclonal)
Caspase 3	Beijing Biosynthesis Biotechnology CO.	bs-2593R	Rabbit (polyclonal)
SOD1	Beijing Biosynthesis Biotechnology CO.	bs-10216R	Rabbit (polyclonal)
Actin	Abcam	ab3280	Mouse(monoclonal)

Table S2. The qRT-PCR primers sequences and the length of the amplified products.

Primer	Sense Primer sequence 5'→3'	Antisense Primer sequence 5'→3'
Caspase3	ATGGAGAACAACAAAACCTCAGT	TTGCTCCCATGTATGGTCTTTAC
REC8	GGTAAAGACCTGCGAGGAAA	GCGGAGAGATAGAGGGAGAA
SYCP1	CTCAAGGAAACCTGTGCTAGAT	TGCACACGAAGTTCCTCAA
SYCP3	AGCAGAGAGCTTGGTCGGG	TCCGGTGAGCTGTCGCTGTC
PRM1	AGCAAAAGCAGGAGCAGATG	CTTGCTATTCTGTGCATCTAG
β-Actin	AGGTCGGTGTGAACGGATTTG	TGTAGACCATGTAGTTGAGGTCA
